# Aberrant DNA Methylation Is Associated with Disease Progression, Resistance to Imatinib and Shortened Survival in Chronic Myelogenous Leukemia

**DOI:** 10.1371/journal.pone.0022110

**Published:** 2011-07-08

**Authors:** Jaroslav Jelinek, Vazganush Gharibyan, Marcos R. H. Estecio, Kimie Kondo, Rong He, Woonbok Chung, Yue Lu, Nianxiang Zhang, Shoudan Liang, Hagop M. Kantarjian, Jorge E. Cortes, Jean-Pierre J. Issa

**Affiliations:** 1 Department of Leukemia, The University of Texas MD Anderson Cancer Center, Houston, Texas, United States of America; 2 Department of Bioinformatics and Computational Biology, The University of Texas MD Anderson Cancer Center, Houston, Texas, United States of America; Clinica Universidad de Navarra, Spain

## Abstract

The epigenetic impact of DNA methylation in chronic myelogenous leukemia (CML) is not completely understood. To elucidate its role we analyzed 120 patients with CML for methylation of promoter-associated CpG islands of 10 genes. Five genes were identified by DNA methylation screening in the K562 cell line and 3 genes in patients with myeloproliferative neoplasms. The *CDKN2B* gene was selected for its frequent methylation in myeloid malignancies and *ABL1* as the target of *BCR-ABL* translocation. Thirty patients were imatinib-naïve (mostly treated by interferon-alpha before the imatinib era), 30 were imatinib-responsive, 50 were imatinib-resistant, and 10 were imatinib-intolerant. We quantified DNA methylation by bisulfite pyrosequencing. The average number of methylated genes was 4.5 per patient in the chronic phase, increasing significantly to 6.2 in the accelerated and 6.4 in the blastic phase. Higher numbers of methylated genes were also observed in patients resistant or intolerant to imatinib. These patients also showed almost exclusive methylation of a putative transporter *OSCP1*. Abnormal methylation of a Src suppressor gene *PDLIM4* was associated with shortened survival independently of CML stage and imatinib responsiveness. We conclude that aberrant DNA methylation is associated with CML progression and that DNA methylation could be a marker associated with imatinib resistance. Finally, DNA methylation of *PDLIM4* may help identify a subset of CML patients that would benefit from treatment with Src/Abl inhibitors.

## Introduction

DNA methylation in promoter-associated CpG islands is a powerful mechanism of gene silencing that is one of the drivers of neoplastic transformation through the inactivation of critical tumor-suppressor pathways [1]. DNA hypermethylation is commonly seen in various types of leukemia including acute myeloid leukemia [2], acute lymphoblastic leukemia [3], chronic lymphocytic leukemia [4], [5], [6], and it has recently been shown to predict outcomes in some patients with myelodysplastic syndrome [7], [8]. Etiologically, chronic myelogenous leukemia (CML) is a homogeneous genetic disease, as it is triggered by the aberrant tyrosine kinase activity of the *BCR-ABL* translocation [9].

Despite genetic homogeneity, there is considerable heterogeneity in the clinical course of CML: it progresses at a varying rate from the chronic phase (CP) to the accelerated phase (AP) and eventually to the blastic phase (BP). Tyrosine kinase inhibitors such as imatinib mesylate (imatinib) are very effective in treating chronic-phase CML but considerably less effective in treating blastic-phase CML [10]. This heterogeneity in disease progression and response to imatinib therapy is likely due to molecular events that follow the initial *BCR*-*ABL* translocation. Aberrant hypermethylation has been previously described in CML [11], [12], [13], [14], [15], [16]. The translocated *ABL1* promoter shows allele-specific de novo methylation early on in the course of the disease, a phenomenon that is unique to CML [14], [17]. A few studies have examined the methylation status of individual tumor-suppressor genes in CML, with results ranging from rare or no hypermethylation (e.g., *SFRP1*, *RASSF1A*) [18], [19] to hypermethylation at progression (e.g., *CALCA*, *CDKN2B*, *EBF2*, *ESR*, *HIC1*, *TFAP2A*, and others) [11], [12], [13], [20]. Hypermethylation of *ATG16L2* gene promoter has been associated with a poor response to imatinib treatment [20]. However, these studies have been limited by the relatively random choice of genes examined, which was based on studies of other malignancies.

Here, we report on the methylation status of a set of 10 genes. Five genes were selected based on our genome wide methylation studies in the K562 leukemia cell line [21] and 3 genes based on our genome wide screening in patients with myeloproliferative neoplasms [22]. We have also included the *CDKN2B* gene, deleted in K562 and frequently methylated myeloid malignancies [23], and the *ABL1* gene as the target of *BCR-ABL* translocation. We found that DNA methylation was strongly associated with disease progression and resistance to imatinib in CML.

## Methods

### Patients and cell line

We examined gDNA from peripheral blood mononuclear cells of 120 patients with CML at various phases (65 in CP, 40 in AP, and 15 in BP) that had been treated at The University of Texas MD Anderson Cancer Center (Houston, TX). The median age was 50 years (range 16–80 years), 79 patients (65%) were male. One set of samples from 30 patients that had been collected between November 1988 and June 1993 was studied to determine the effect of DNA methylation on CML progression prior to the imatinib era (i.e., imatinib-naïve patients). Most of these patients had been treated with interferon-alpha–based regimens. Another set of samples from 90 patients treated in the imatinib era was obtained between July 2001 and November 2004. Of these 90 patients, 30 were imatinib-responsive (27 in CP, 2 in AP and 1 in BP), 50 were imatinib-resistant (10 in CP, 28 in AP and 12 in BP), and 10 were imatinib-intolerant (6 in CP, 4 in AP and 0 in BP). None of the patients had been previously treated with hypomethylating drugs. Clinical and hematological data of the patients are summarized in Table 1. For normal controls, peripheral white blood cells (WBC) were collected from 22 healthy volunteers (18–53 years of age). The Institutional Review Board at MD Anderson approved all protocols, and all patients gave informed consent for the collection of residual tissues as per institutional guidelines and in accordance with the Declaration of Helsinki.

**Table 1:** Characteristics of the patients.

Parameter	Pre-imatinib	Imatinib era
Sample dates	11/1988–06/1993	07/2001–11/2004
Total patients	30	90
Age, years; median (range)	42 (16–69)	54 (23–80)
Males	20 (67%)	58 (64%)
CML stage		
chronic	21	43
accelerated	7	34
blastic	2	13
Imatinib status		
naïve	30	0
responsive	N/A	30
resistant	N/A	50
intolerant	N/A	10
Hematological parameters		
WBC, 10∧3/uL; median (range)	119 (2–366)	23 (3–317)
Peripheral blood blasts, %; median (range)	2 (0–92)	1 (0–99)
Peripheral blood basophils, %; median (range)	2 (0–22)	3 (0–36)
Hemoglobin, g/dL; median (range)	11.4 (5.8–15.2)	10.9 (6.8–16.6)
Platelets, 10∧3/uL; median (range)	259 (50–1205)	222 (10–1245)
Bone marrow blasts, %; median (range)	2 (0–91)	2 (0–94)
Bone marrow basophils, %; median (range)	2 (0–11)	3 (0–27)

The leukemia cell line K562 used in this study was obtained from the American Type Culture Collection (Manassas, VA, USA).

### Methylated CpG island amplification microarray (MCAM) analysis

We used gDNA from the CML-derived K562 cell line [24] and, as a control, a DNA pool made from WBC of 4 healthy donors. In separate MCAM experiments (data not shown), we found minimal differences in DNA methylation of the analyzed CpG sites between CD34^+^ bone marrow cells and unsorted WBC, suggesting that the chosen control was appropriate for MCAM analysis. Methylated CpG island amplification (MCA) was performed as described previously [25]. Amplicons from the K562 cell line were labeled with the Cy5 dye and cohybridized against amplicons from WBC control labeled with the Cy3 dye on Agilent Technologies 4×44 K custom DNA microarrays (Agilent, Santa Clara, CA) as described previously [2]. MCAM for K562 was performed as a single array experiment. Fluorescence signals were lowess normalized and trimmed averages of normalized log_2_ ratios were calculated for amplicons covered by multiple probes. Hypermethylation was defined as normalized log_2_ ratio of Cy5/Cy3 fluorescence greater than 1 (equivalent to 2-fold and higher K562/WBC signal intensity). MCAM has been extensively validated by independent bisulfite-based methods showing the sensitivity of 88% and the specificity of 96% [26]. Enrichment for Polycomb targets was performed by comparing genes differentially methylated in the K562 cell line with the list of targets of H3K27 trimethylation in human embryonic stem cells [27] that were present on our array. The effect of methylation on gene expression was assessed using GNF1H data sets [28] available from the Gene Expression Omnibus (http://www.ncbi.nlm.nih.gov/geo/).

### DNA methylation analysis by bisulfite pyrosequencing

We isolated gDNA from peripheral blood mononuclear cells after Ficoll separation using standard phenol-chloroform extraction. We selected 10 genes and the LINE-1 repetitive element for quantitative analysis of DNA methylation by bisulfite pyrosequencing as described previously [2], [29]. Our pyrosequencing assays interrogated 2–6 adjacent CpG sites close to gene transcription start site (Table 2). Methylation status of consecutive CpG sites has a high concordance in regions spanning several hundred bases [30]. Therefore, we used mean values from all pyrosequenced CpG sites as a measure of methylation of a given gene. For each assay, we determined the range of normal values by measuring the DNA methylation levels in 18–22 healthy controls. Methylation values exceeding the maximum value detected in normal controls were considered abnormal. This criterion was more stringent that the 95% confidence interval. Genomic location of the bisulfite pyrosequencing assays and the number of investigated CpG sites in each assay are shown in Table 2. The sequences of PCR primers and annealing temperatures are listed in Table S1.

**Table 2:** Genomic location of bisulfite pyrosequencing assays.

Gene	Location of pyrosequencing target (hg18)	Distance from TSS	CpG sites in assay
*ABL1*	chr9:132,700,627–132,700,645	−24 to −14	3
*CDH13*	chr16:81,218,151–81,218,189	+74 to +102	6
*CDKN2B*	chr9:21,999,154–21,999,185	+127 to +147	4
*DPYS*	chr8:105,548,436–105,548,480	−27 to +17	4
*NPM2*	chr8:21,938,232–21,938,267	−66 to −41	6
*OSCP1*	chr1:36,688,776–36,688,815	−37 to −20	5
*PDLIM4*	chr5:131,621,029–131,621,064	−221 to −192	6
*PGR-A*	chr11:100,504,896–100,504,924	+261 to +289	4
*PGR-B*	chr11:100,505,573–100,505,605	+860 to +892	4
*TFAP2E*	chr1:35,811,437–35,811,445	−121 to −113	2

### Statistical analysis

Lowess normalization and analysis of MCAM data were performed as described [26]. To analyze enrichment for Polycomb targets the chi-square test was used, and odds ratio for enrichment was calculated. Pathways affected by aberrant methylation of multiple genes were identified with Ingenuity Pathway Analysis software (Ingenuity Systems, Redwood City, CA). We used the Wilcoxon signed rank nonparametric test to compare the expression of genes in K562 and normal white blood cells. We used the Spearman nonparametric correlation test to compare bisulfite pyrosequencing methylation data between individual genes. We used the chi-square and Fisher's exact tests to compare DNA methylation data with clinical parameters. We performed multivariate analysis using Cox's regression and forward stepwise likelihood ratio model to find independent prognostic variables. We used Kaplan-Meier logrank tests to calculate and generate overall survival curves for independent variables. Two-tailed *P* values of 0.05 or less were considered statistically significant. We used GraphPad Prism 5 (GraphPad Software, La Jolla, CA) and PASW Statistics 17.0 to perform statistical analyses.

## Results

### MCAM analysis

**Figure 1:**
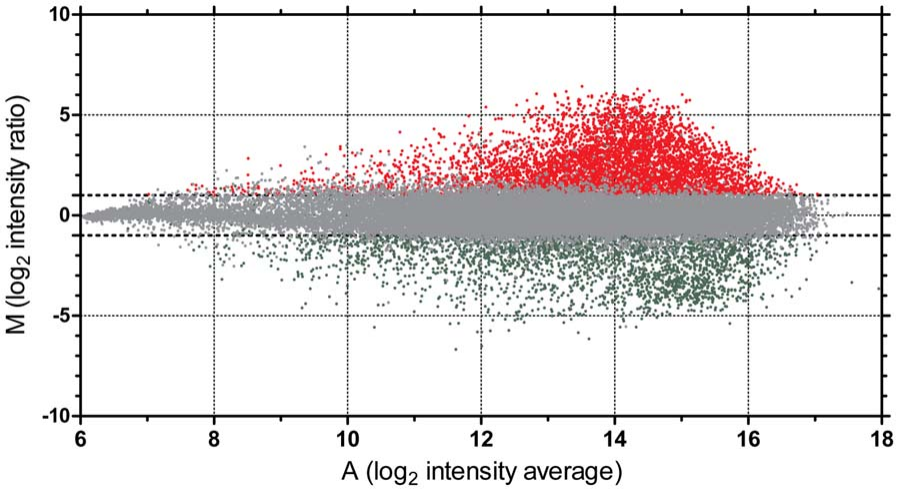
DNA methylation in the K562 leukemia cell line analyzed by MCAM. Red dots correspond to hypermethylated DNA fragments, green dots show hypomethylated fragments and grey dots depict no significant changes when compared to normal white blood cells. Horizontal axis, A, average log_2_ of signal intensity; vertical axis, M, log_2_ of Cy5/Cy3 normalized fluorescence ratio of K562/normal white blood cells.

**Figure 2:**
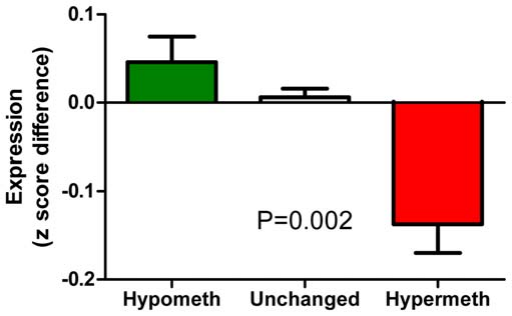
DNA methylation and gene expression in the K562 leukemia cell line. Hypermethylation is associated with lower expression. Bars from left to right, DNA fragments hypomethylated (green), unchanged (grey), and hypermethylated (red) in K562 located within 500 bases of gene transcription start sites. The vertical axis shows differences in z-score normalized log values of gene expression between the K562 cell line and normal white blood cells. Error bars show S.E.M.

We observed hypermethylation in the K562 cell line in 4,138 of 27,890 (15%) total CpG sites analyzed by MCAM (Figure 1). When we focused on CpG sites within 500 bp from TSS, we detected hypermethylation in 1,014 of 7,246 (14%) RefSeq autosomal genes analyzed. The complete list of methylated genes is provided in Table S2. The methylated genes showed an enrichment for targets of Polycomb (PCG) silencing in embryonic stem cells. The microarray we used for MCAM could detect methylation status of 5,143 autosomal genes with available information on PCG targeting in ES cells [27]. Among 688 genes showing hypermethylation, 178 genes (25.8%) were PCG targets. Among 4,455 unmethylated genes, only 491 genes (11.0%) were PCG targets, odds ratio 2.8 (95% confidence interval 2.3 to 3.4, *P*<.0001, Table S3). Analysis of pathways affected by methylation of multiple genes revealed a significant enrichment for genes involved in cellular development (201 genes, *P*<.01), cell death (190 genes, *P*<.01), and gene expression (149 genes, *P*<.01). The list of gene categories significantly affected by DNA methylation is shown in Table S4. Based on the Gene Expression Omnibus GNF1H data sets, the hypermethylated genes as detected by MCAM also had a significantly lower expression in the K562 cell line than in normal white blood cells (*P* = .002; Figure 2).

### Bisulfite pyrosequencing analysis

We selected 10 genes for quantitative analysis of DNA methylation by bisulfite pyrosequencing in CML patients. *DPYS*, *NPM2*, *OSCP1*, *PDLIM4* and *TFAP2E* genes were found hypermethylated, and *CDKN2B* (p15^INK4B^) homozygously deleted in the K562 leukemia cell line. *ABL1* is the subject of *BCR-ABL* translocation and is methylated exclusively on the Philadelphia chromosome. *CDH13*, *PGR*-A and B isoforms were identified by a genome wide screening as hypermethylated in patients with myeloproliferative neoplasms [22]. We also analyzed methylation of the LINE-1 repetitive element, as a surrogate marker of global genomic methylation [31]. A summary of the results of the DNA methylation analysis in 120 patients with CML is shown in Table 3. The most frequently methylated genes in more than 70% patients across all CML stages were *ABL1*, *CDH13* and *NPM2*. Interestingly, about half of CML patients also showed an increase in methylation of the LINE-1 repetitive element above the normal range. However, only 4 patients (3%) showed LINE-1 methylation below the normal range. The differences in LINE-1 methylation between individual stages of CML were not significant (Table S5).

**Table 3:** DNA methylation in CML patients.

Gene	Cutoff*	Patients methylated (%)				P value**
	(% methylation)	CP (n = 64)	AP (n = 41)	BP (n = 15)	Total (n = 120)	
*ABL1*	9	52 (81)	37 (90)	14 (93)	103 (86)	NS
*CDH13*	8	47 (73)	34 (83)	14 (93)	95 (79)	NS
*CDKN2B*	7	2 (3)	7 (17)	4 (27)	13 (11)	0.009
*DPYS*	14	30 (47)	23 (56)	12 (80)	65 (54)	NS
*NPM2*	4	45 (70)	32 (78)	12 (80)	89 (74)	NS
*OSCP1*	4	10 (16)	19 (46)	7 (47)	36 (30)	0.001
*PDLIM4*	33	8 (13)	12 (29)	5 (33)	25 (21)	NS
*PGRA*	6	36 (56)	33 (80)	10 (67)	79 (66)	0.038
*PGRB*	11	25 (39)	24 (59)	13 (87)	62 (52)	0.002
*TFAP2E*	20	32 (50)	32 (78)	11 (73)	75 (63)	0.010

*The highest methylation value observed in normal controls.

**Chi-square test.

### DNA methylation is increased in advanced CML

Five of the 10 studied genes showed a significant methylation increase in CML progressed to AP or BP stages. These included *CDKN2B*, *OSCP1*, *PGRA*, *PGRB* and *TFAP2E* genes (Table 3). We next calculated the sums of methylated and unmethylated genes in individual patients and analyzed the proportions of methylated and unmethylated genes in CML stages. On average, 4.5 genes were methylated in CP, 6.2 genes in AP and 6.8 genes in BP (Table 4). The increase in the number of methylated genes in advanced stages was statistically significant (*P*<.0001, chi-square test). Of 64 patients in CP, 1 patient (2%) had no hypermethylated gene and 21 patients (33%) had more than 5 hypermethylated genes. Of 41 patients in AP, no patient (0%) had zero hypermethylated genes and 25 patients (61%) had more than 5 hypermethylated genes. Of 15 patients in BP, no patient (0%) had no hypermethylated gene and 12 patients (80%) had more than 5 hypermethylated genes. There was no significant difference in overall survival between 35 CP patients with 0–4 hypermethylated genes and 29 CP patients with 4–10 hypermethylated genes. Methylation levels showed positive correlations between individual genes (Table S6). Methylation of the *DPYS* gene was signicantly correlated with 6 other genes, suggesting this gene may be a part of a potential hypermethylator phenotype and a shared etiology for increased methylation in a subset of affected cases. On the other hand, the *PDLIM4* gene stood out showing no significant correlation with any of the other analyzed genes.

**Table 4:** Methylation and CML stage.

Stage	Sum of methylated genes	Sum of unmethylated genes	P value	Average of methylated genes per patient
CP	287	353		4.5
AP	253	157	<0.0001	6.2
BP	102	48		6.8

### DNA methylation is increased in imatinib resistant and intolerant patients

To assess a possible epigenetic component of imatinib resistance, we compared DNA methylation in patients responsive, intolerant and resistant to imatinib. Patients in CP that were responsive to imatinib had on average 3.9 methylated genes, while patients intolerant or resistant to imatinib had 6.3 or 5.4 methylated genes, respectively (*P* = .0004, chi-square test). A similar increase in the number of methylated genes in patients intolerant or resistant to imatinib was also observed in AP, but not in BP (Table 5). When we analyzed individual genes, the frequency of aberrant methylation of *OSCP1* and *NPM2* was significantly higher in resistant and intolerant patients than in responsive patients. In the subset of CP patients, aberrant methylation of *OSCP1* was seen only in the patients that were resistant or intolerant to imatinib (Table S7).

**Table 5:** Methylation and response to imatinib.

Stage	Imatinib response	Sum of methylated genes	Sum of unmethylated genes	P value	Average of methylated genes per patient
All stages	Responsive	120	180	<0.0001	4.0
	Intolerant	62	38		6.2
	Resistant	318	182		6.4
CP	Responsive	104	166	0.0004	3.9
	Intolerant	38	22		6.3
	Resistant	54	46		5.4
AP	Responsive	8	12	0.0498	4.0
	Intolerant	24	16		6.0
	Resistant	186	94		6.6
BP	Responsive	8	2	NS	8.0
	Intolerant	0	0		N/A
	Resistant	78	42		6.5

We could not assess the relationship between resistance to imatinib, DNA methylation and *ABL1* mutations, since we had data on *ABL1* mutational status for 10 patients only. Two patients were responsive to imatinib, negative for *ABL1* mutations. They had 2 and 5 genes hypermethylated, respectively; however, they did not show hypermethylation of *OSCP1* or *PDLIM4*. One patient was intolerant to imatinib and negative for *ABL1* mutations. The patient had 6 hypermethylated genes, however, no hypermethylation of *OSCP1* or *PDLIM4*. Seven patients with known mutational status of *ABL1* were resistant to imatinib. Three of them had mutations in *ABL1*, F317L, F359V and a mutation in codon 355, respectively. These patients had 4, 6 and 6 hypermethylated genes, respectively. None of these patients showed hypermethylation of the *OSCP1* or *PDLIM4* gene. Four imatinib-resistant patients were negative for *ABL1* mutations. They had 5, 7, 8 and 9 hypermethylated genes. Interestingly, three of these 4 patients had hypermethylated the *OSCP1* gene and two had also hypermethylated the *PDLIM4* gene.

### DNA methylation and outcome

**Figure 3:**
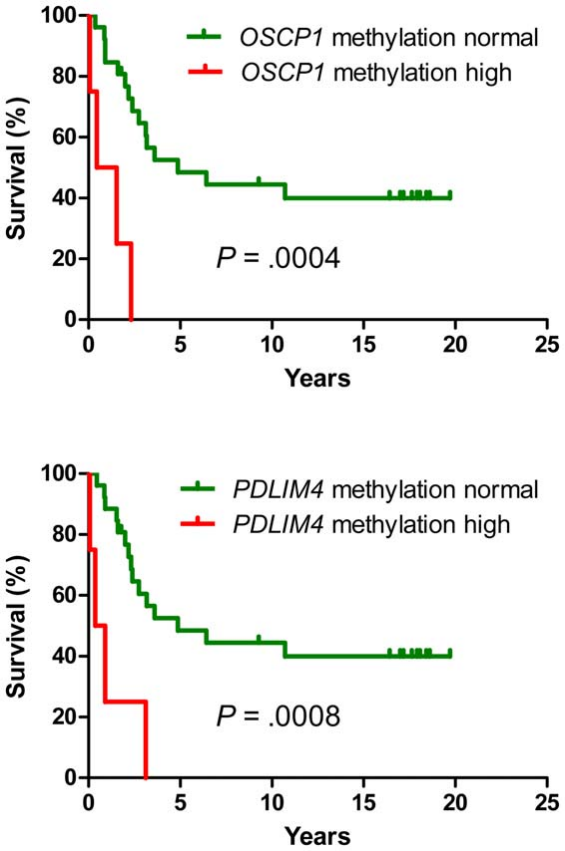
Methylation of *OSCP1* and *PDLIM4* in CML patients from the pre-imatinib era. Hypermethylation of *OSCP1* (top) and *PDLIM4* (bottom) is associated with shortened survival. Green line, methylation within normal range. Red line, hypermethylation.

**Figure 4:**
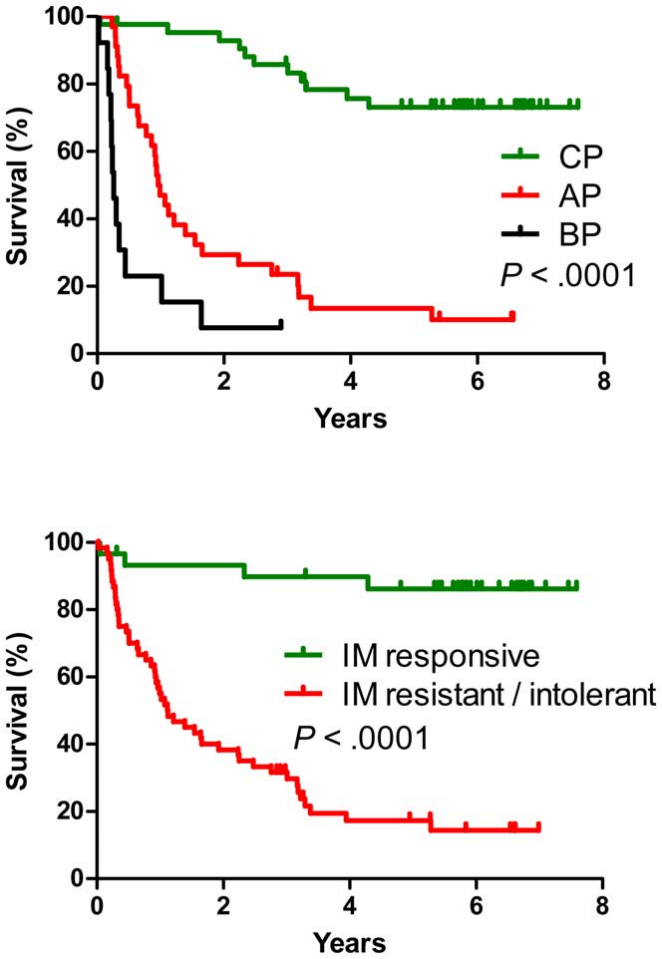
Advanced stage of CML and resistance or intolerance to imatinib are associated with shortened survival. Top graph, green, chronic phase; red, accelerated phase; black, blastic phase. Bottom graph, green, imatinib-responsive patients; red, patients resistant or intolerant to imatinib. The figure shows 90 CML patients (43 CP, 34 AP, 13 BP) from the imatinib era.

**Figure 5:**
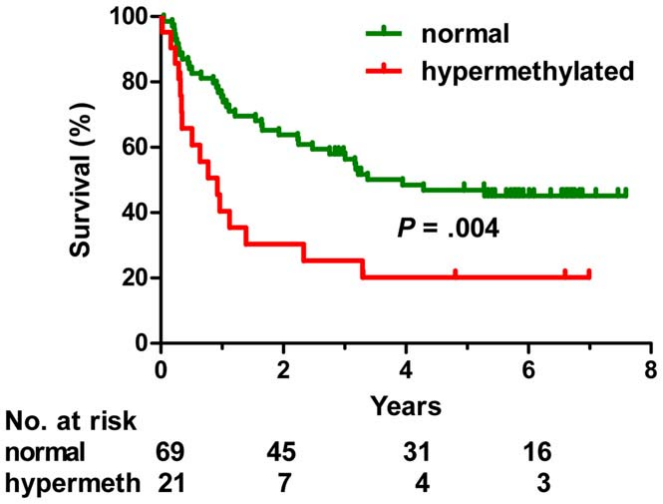
Hypermethylation of the *PDLIM4* gene is associated with shortened survival in CML patients treated in the imatinib era. Green line, methylation within normal range. Red line, hypermethylation. The figure shows 90 CML patients (43 CP, 34 AP, 13 BP) from the imatinib era.

Having established that DNA methylation is increased in more advanced stages of CML and in patients resistant or intolerant to imatinib, we used multivariate analysis to examine if aberrant methylation is an independent prognostic variable. Since imatinib treatment changed radically the outcome of CML, we analyzed separately the groups of patients treated before and in the imatinib era (Table 6). Hypermethylation of *OSCP1* and *PDLIM4* genes were negative risk factors in the pre-imatinib era patients (hazard ratio 9.6 and 8.1, respectively, *P* = 0.001). Median survival was 1 year for *OSCP1*-methylated and 0.6 years for *PDLIM4*-methylated patients, while the patients with no methylation had median survival of 4.9 years (Fig. 3). Overall survival of the second group of 90 patients diagnosed and treated after 2001 (imatinib era) is shown in Fig. 4 and Fig. 5. Advanced CML stage (hazard ratio 4.0, *P*<.001), resistance or intolerance to imatinib (hazard ratio 5.4, *P* = .002) and hypermethylation of the *PDLIM4* gene (hazard ratio 2.7, P = 0.003) were independent prognostic variables (Table 6 and Figure 4). Median survival of the patients with hypermethylated *PDLIM4* was 0.9 years while it was 3.9 years in the patients where *PDLIM4* methylation was within the normal range (Figure 5). When we restricted the analysis to 30 imatinib-responsive patients only, 3 deaths were observed in 26 patients with *PDLIM4* methylation within normal range, while 1 death was observed in 4 patients with hypermethylated *PDLIM4*. However, the difference in survival was not statistically significant, possibly due to small number of patients and events. Methylation status of the LINE-1 repetitive element did not show any association with the outcome or the stage of the disease.

**Table 6:** Multivariate analysis of DNA methylation and clinical data.

Group of patients	Independent variable	Hazard ratio	P value
Pre-imatinib, n = 30	*OSCP1* methylation	9.6	0.001
	*PDLIM4* methylation	8.1	0.001
Imatinib era, n = 90	Disease stage	4.0	<0.001
	Imatinib resistance/intolerance	5.4	0.002
	*PDLIM4* methylation	2.7	0.003

## Discussion

In this study, we have shown that aberrant DNA methylation of multiple genes characterizes advanced stages of CML and the disease when resistant to imatinib. Our interpretation is that the disease progression is associated with epigenetic changes including aberrant DNA methylation. We speculate that epigenetically mediated silencing of genes involved in drug transport may also affect the responsiveness of leukemic cells to imatinib. Given that CML starts as a genetically homogeneous disease, one can hypothesize that disease progression and clinical heterogeneity in CML are related to epigenetic factors including DNA hypermethylation. Our data extend previous observations on DNA methylation in CML and identify multiple new biomarkers in the disease. Of the genes specifically studied here, some may in fact contribute to the pathophysiology of disease progression. For instance, *PDLIM4*, also known as RIL, is a LIM domain protein that has tumor-suppressor and pro-apoptotic properties. We have previously described a significant correlation between methylation and silencing of this gene [32]. *PDLIM4* was found hypermethylated and silenced in prostate cancer. Restoration of its expression suppressed tumor growth in xenografts [33]. A recent report suggests that PDLIM4 is important for inactivation of Src and that epigenetic silencing of *PDLIM4* may contribute to aberrant activation of Src in cancer [34]. Hypermethylation of *PDLIM4* in our sets of CML patients had a negative prognostic impact independent of the response to imatinib. We suggest that CML patients with epigenetically silenced *PDLIM4* may particularly benefit from treatment with Src/Abl inhibitors.

*TFAP2E*, or transcription factor AP-2 epsilon, is a gene that we have found to have a potential tumor-suppressor function (and correlation between methylation and expression) [35]. *CDH13*, or H-cadherin, is a cell adhesion molecule with tumor-suppressor properties and an established correlation between methylation and expression [15]. Methylation of *CDKN2B* or the *p15* tumor suppressor gene is frequently reported in myeloid malignancies [36]. However, *CDKN2B* was methylated only in 11% of the CML patients in our study, and its methylation did not show an association with shortened survival.

Aberrrant methylation of other genes likely represents passenger epigenetic defects that reflect the pressures to increase promoter DNA methylation in neoplastic cells. *NPM2*, or nucleophosmin 2, is involved in forming nucleolus-like bodies in oocytes [37]. It is aberrantly methylated in patients with acute myeloid leukemia [2] and melanoma [38]. Progesterone receptor methylation has been reported in leukemia [2] and solid cancers [39], [40], [41], [42]. The role of *DPYS*, or dihydropyrimidinase, in the hematopoietic system is currently unknown, and it may simply be a marker of methylation defects in CML as is the case in prostate and breast cancer [35].

Methylation of the *OSCP1* gene (formerly known as *C1orf102* or *NOR1*) and its strong association with resistance to imatinib is intriguing. Aberrant methylation of *OSCP1* has been shown in nasopharyngeal carcinoma [43] and acute myeloid leukemia [2]. This gene codes for an organic solute carrier protein with broad substrate specificity [44]. The gene product may be involved in the transport of imatinib to target cells and its silencing may thus contribute to imatinib resistance.

We found strong concordant methylation for several of the genes tested, which cannot be explained simply by phase-specific methylation as concordant methylation also occurred within each phase. The genes involved do not share features, such as structure, chromosomal location, or function, and so this concordant methylation was likely caused by patient-specific pressures to increase DNA methylation, a phenomenon akin to the CpG island methylator phenotype described in colon cancer [45]. The causes of this phenotype remain unknown, and whether the same factors that lead to it in solid tumors are involved in leukemia pathogenesis or progression remains to be determined.

Complex changes of DNA methylation in cancer can be summarized as focal hypermethylation of promoter CpG islands and global hypomethylation elsewhere, including repetitive elements [46]. We have previously shown that methylation of the LINE-1 repetitive element in cancer and leukemia was highly variable [47]. In this paper, we found that LINE-1 was methylated above the normal range in 55% of CML patients and only 3% of patients showed LINE-1 hypomethylation. This is in contrast with reports of LINE-1 hypomethylation and transcriptional activation in CML and progressive hypomethylation in the advanced phase of the disease [48], [49].

Further analysis of the data on higher DNA methylation of *OSCP1* and other genes in imatinib-resistant patients is warranted. It is possible that gene silencing provides an alternative to *BCR-ABL* mutations in conferring imatinib resistance. Indeed, only about 50% of imatinib resistance can be conclusively traced to acquired mutations [50]. It will therefore be worthwhile to study mutations and methylation simultaneously and determine whether there is an inverse correlation between the two events, and whether it is relevant to resistance to other tyrosine kinase inhibitors. It is also interesting to consider the fact that DNA methylation can be partially reversed by treatment with decitabine or azacitidine. Decitabine has demonstrated single-agent activity in CML [29], and a combination of decitabine and imatinib has shown a promising response rate in AP and BP [51]. Given that many patients with blastic-phase CML continue to die of their disease, such drug combinations may be relevant even after therapy with second-generation tyrosine kinase inhibitors.

## Supporting Information

Table S1 **Bisulfite PCR and pyrosequencing primers.**
(XLS)

Table S2 **List of genes methylated in the K562 cell line.** Genomic coordinates are based on the March 2006 Assembly (NCBI36/hg18). XmaFrag_Start, coordinate of the 5′ *Xma*I site. XmaFrag_End, coordinate of the 3′ *Xma*I site. Gene1, gene with the start site closest to the 5′ *Xma*I site. Gene2, gene with the start site closest to the 3′ *Xma*I site. Distance2TSS_Gene1, distance to transcription start site closest to the 5′ *Xma*I site. Distance2TSS_Gene2, distance to transcription start site closest to the 3′ *Xma*I site. Signal_Intensity_Average, average log2 of signal intensity of probes covering the *Xma*I fragment. Log2_Ratio_K562vsControl, average log2 ratio of Cy5/Cy3 signal intensity of probes covering the *Xma*I fragment.
(XLS)

Table S3 **Genes methylated in the K562 leukemia cell line are enriched for targets of Polycomb in ES cells.**
(XLS)

Table S4 **Gene categories affected by DNA methylation in K562.**
(XLS)

Table S5 **LINE-1 methylation.**
(XLS)

Table S6 **Spearman correlation between methylation of different genes.** In each cell, the top number is the correlation coefficient r. The bottom number is a *P* value. To correct for multiple comparisons, *P*<.001 was considered significant.
(XLS)

Table S7 **Methylation of individual genes and the response to imatinib.**
(XLS)
